# Epilepsy in Cerebral Palsy: Unraveling Prevalence, Risk Factors, and Subtype Associations in a Large-Scale Population Study

**DOI:** 10.3390/medicina60111809

**Published:** 2024-11-04

**Authors:** Reem Abdullah Alyoubi, Ahmed Abu-Zaid

**Affiliations:** 1 Department of Pediatrics, Faculty of Medicine, King Abdulaziz University, Jeddah 22254, Saudi Arabia; 2 Department of Biochemistry and Molecular Medicine, College of Medicine, Alfaisal University, Riyadh 11533, Saudi Arabia

**Keywords:** cerebral palsy, epilepsy, National Inpatient Sample, prevalence, risk factors

## Abstract

*Background and Objective*: Cerebral palsy (CP) constitutes a group of enduring movement disorders arising from non-progressive brain damage, often accompanied by epilepsy. This study aims to explore the prevalence of epilepsy in CP patients, dissecting demographic characteristics, healthcare parameters, and nuanced risk factors. *Materials and Methods*: The study employed the National Inpatient Sample (NIS) database (2016–2019, four years). CP patients were identified through ICD-10 codes, excluding non-CP patients and those with missing values. Baseline characteristics of CP patients, such as age, subtype distribution, and types of epilepsy, were tabulated, and disparities were assessed using the chi-squared test. Univariate and multivariable logistic regression analyses were conducted to examine predictors of epilepsy according to CP subtypes and infant-related conditions. Data were presented as odds ratios (OR) with corresponding 95% confidence intervals (CI). *Results*: In this comprehensive analysis of 88,138 CP patients, 44,901 with epilepsy and 43,237 without epilepsy, disparities between those with and without epilepsy were uncovered, revealing distinct demographic patterns and healthcare characteristics. Spastic quadriplegic CP showed the strongest association with epilepsy (adjusted OR = 2.37, 95% CI [2.29–2.45], *p* < 0.0001), underscoring the importance of subtype-specific considerations. Perinatal infection emerged as a noteworthy risk factor for epilepsy development (adjusted OR = 1.61, 95% CI [1.17–2.23], *p* = 0.004). *Conclusions*: The study provides nuanced insights into the prevalence and associations of epilepsy in CP patients. Specific CP subtypes and perinatal factors play pivotal roles in epilepsy risk. These findings offer a foundation for tailored clinical management and support services, addressing the complex needs of individuals with CP and epilepsy.

## 1. Introduction

Cerebral palsy (CP) is a group of permanent movement and posture disorders that result from non-progressive damage to the developing brain, particularly in areas responsible for motor control. This damage can occur during fetal development or in the early years of life, impairing muscle coordination, balance, and posture, leading to limitations in a person’s ability to move and perform daily activities. The resulting symptoms can appear in varying degrees of severity, showcasing the complex challenges individuals with CP pose to both social and healthcare systems. Different presentations of CP include spasticity, dyskinesia, ataxia, and mixed forms, each contributing to distinct motor impairments [1,2,3].

Epilepsy is prevalent in a notable portion of the CP population, with prevalence rates ranging from 15% to 90%. However, most estimates tend to cluster around 35% to 41% [4,5]. The likelihood of developing epilepsy in the CP population is linked to the specific CP subtype, with a higher incidence observed in individuals with tetraplegia and the ataxic form of CP [6]. Recent research, drawing from population data, underscores that approximately 50% of cases exhibit epilepsy remission with antiseizure medication (ASM), placing special emphasis on the relevance of CP subtypes. Notably, spastic quadriplegia is linked with a remission rate as low as 20% [5].

Understanding the relationship between CP and epilepsy is crucial not only for clinical management but also for improving the quality of life for affected individuals [7]. The coexistence of these conditions can significantly complicate treatment regimens, as seizures can exacerbate motor impairments and hinder rehabilitation efforts [8]. For instance, the unpredictability of seizure activity may lead to increased caregiver stress and necessitate adjustments in therapy schedules, potentially impeding the overall progress of motor skills and functional independence. This interplay between epilepsy and motor function highlights the need for integrated care approaches that address both conditions simultaneously.

Furthermore, the psychosocial implications of epilepsy in individuals with CP—such as increased stigma, anxiety, and social isolation—can profoundly affect their overall well-being and development [9,10]. Children with these comorbidities may face challenges in social interactions, as their conditions can lead to misunderstandings or negative perceptions from peers and adults. This stigma can result in diminished self-esteem and reluctance to engage in activities that promote socialization and inclusion, such as sports or group learning environments. The added layer of anxiety surrounding the unpredictability of seizures can exacerbate these issues, leading to a cycle of withdrawal and further isolation [9,10].

The impact of epilepsy on mental health should not be underestimated. Individuals with CP who experience seizures may be at an increased risk for anxiety and depression, which can further complicate their rehabilitation and daily functioning. Mental health support is essential for these patients, as addressing emotional well-being can enhance their capacity to cope with the challenges posed by both CP and epilepsy. By fostering a holistic approach that includes mental health resources, caregivers and healthcare providers can create a supportive environment that encourages resilience and promotes better quality of life [11,12].

Numerous studies have investigated predictors and risk factors associated with epilepsy in children with CP [13,14,15,16,17,18,19]. These factors span preconception and prenatal considerations, including maternal age, diseases, reproductive history, arterial hypertension, infections, preterm contractions, and abruptio placentae [6]. Perinatal events such as prematurity, low birth weight, and low APGAR score, along with degree of intellectual or developmental disabilities, may also contribute [15,20]. Notably, epilepsy cases in both adults and children may lack an obvious cause, emphasizing that the presence of these factors does not guarantee the development of epilepsy in children with CP [21].

In this study, we aimed to perform a comprehensive investigation using a substantial sample size from the National Inpatient Sample (NIS) and encompassing thousands of patients to provide valuable insights into the prevalence of epilepsy as well as potential risk factors and predictors for its development in CP patients. This first-ever expansive study utilizing the NIS database aims to elucidate the intricate associations between various CP subtypes and the risk of epilepsy, with the ultimate goal of guiding the development of targeted interventions and support services. By employing both univariate and multivariable analyses, we present our findings in tables, allowing for a clearer understanding of the data aligned with our specific objectives. This structured approach enhances the ability to identify key trends and associations that may inform clinical practices and future research directions.

## 2. Methods

We utilized the NIS database as the primary data source, and data spanning a 4-year period from 2016–2019 were consolidated into a single STATA 15 file for analysis. The NIS database is derived from the Healthcare Cost and Utilization Project (HCUP), which is a key initiative of the Agency for Healthcare Research and Quality (AHRQ) [22]. NIS is the largest publicly available database of inpatient admissions in the United States. It provides a representative sample of hospital stays across various states, capturing information on demographic, clinical, and outcome variables. The NIS is designed to facilitate research on hospital utilization, patient outcomes, and healthcare trends, making it a valuable resource for policymakers and researchers. Data are collected from a diverse range of hospitals, allowing for analyses that reflect the broader U.S. healthcare system. By enabling insights into patterns of care and the effectiveness of treatments, the NIS plays a crucial role in advancing healthcare research.

CP patients were identified by searching for the specified codes in diagnosis variables according to the International Classification of Diseases, 10th Edition (ICD-10). The inclusion criteria comprised all CP patients with an assigned ICD-10 code and complete baseline characteristic information. The exclusion criteria encompassed all non-CP patients, as well as CP patients with any missing baseline characteristic information. Detailed codes used in the study for CP, epilepsy, and baseline characteristics are described in Supplementary Table S1. The selection of our CP patient population was based entirely on the availability of related ICD-10 codes. If the codes were available, the patients were included as listed in Supplementary Table S1; otherwise, they were regarded as non-CP and excluded. Missing values were not handled, as patients with any missing information were automatically excluded. In this research, our CP population was further divided into two cohorts for comparison: (i) CP with epilepsy, and (ii) CP without epilepsy.

Baseline characteristics of CP patients, with and without epilepsy, were tabulated, including age, sex, race, primary expected payer, ZIP income quartile, types of CP, types of epilepsy, and various hospital characteristics (bed size, teaching status, and location). The chi-squared (χ^2^) test was employed to assess the disparities in baseline characteristics, particularly categorical variables, between patients with epilepsy and those without it. Inferential statistics, encompassing univariate and multivariable logistic regression analyses, were conducted to examine predictors of epilepsy according to CP subtypes and infant-related conditions. Data were presented as odds ratios (OR) with corresponding 95% confidence intervals (CI). Significance was determined by a lack of overlap with 1 and a *p*-value < 0.05. Adjustment in multiple regression was carried out for significant variables in the univariate analysis and baseline characteristics of the population (age, sex, race, hospital location, hospital teaching status, primary payer, and income).

Notably, this study utilized de-identified, publicly available data and accordingly did not require Institutional Review Board (IRB) approval.

## 3. Results

### 3.1. Summary of Baseline Characteristics of CP Patients

We included 88,138 CP patients: 44,901 with epilepsy and 43,237 without epilepsy. Table 1 presents a comprehensive overview of the demographic and healthcare characteristics among the included CP patients, distinguishing between those with epilepsy and those without. Overall, our analysis underscored some significant disparities between CP patients with and without epilepsy. Notably, CP patients with epilepsy tended to be significantly younger, with a mean age of 28.40 ± 20.5 years compared to 39.81 ± 23.03 years for CP patients without epilepsy. Both groups showed a balanced gender distribution, with males comprising 56.24% of the overall CP population. The distribution of CP cases remained consistent across the years 2016–2019. Spastic quadriplegic CP was the predominant specified subtype in the overall CP population (22.42%), and its prevalence was significantly higher in CP patients with epilepsy (30.13%) compared to CP patients without epilepsy (14.41%). Nearly 60% of the CP patients had an unspecified type of CP. Additionally, three-quarters of the CP patients with epilepsy (75.04%) had an unspecified type of epilepsy. Furthermore, racial composition differed, with a higher percentage of white patients observed in the non-epilepsy group (68.79%) compared to the epilepsy group (58.91%). Additionally, a substantial divergence existed in the primary expected payer distribution, revealing that non-epilepsy group has a notably higher proportion covered by Medicare (46.97%) compared to the epilepsy group (31.73%). Despite these marked differences, the distribution of ZIP income quartiles remained relatively consistent between the two groups.

### 3.2. Association Between Epilepsy and CP Subtypes Among CP Patients

Table 2 provides insights into the predictive risk of epilepsy in CP patients based on different CP subtypes. The univariate analysis revealed substantial variations in the risk of epilepsy across CP subtypes. “Unspecified” CP subtype showed the lowest risk of epilepsy (OR = 0.57, 95% CI [0.55–0.58], *p* < 0.0001), whereas the “spastic quadriplegic” CP subtype exhibited the highest risk of epilepsy (OR = 2.56, 95% CI [2.48–2.65], *p* < 0.0001). In the multivariable analysis, the “spastic quadriplegic” CP subtype was associated with the highest risk of epilepsy (OR = 2.37, 95% CI [2.29–2.45]; *p* < 0.0001), whereas the “ataxic” CP subtype was associated with the lowest risk (OR = 0.60, 95% CI [0.48–0.75], *p* < 0.0001).

### 3.3. Association Between Epilepsy and Infant-Related Conditions Among CP Patients

Table 3 elucidates the infant-related risk factors associated with epilepsy in individuals diagnosed with CP. The univariate analysis identified several factors significantly linked to the occurrence of epilepsy. Perinatal infection exhibited a substantial association (OR = 2.64, 95% CI [1.94–3.61], *p* = 0.0001) which was reinforced through multivariable analysis (OR = 1.61, 95% CI [1.17–2.23], *p* = 0.004). The study did not find significant associations with perinatal cardiovascular respiratory disorders and bronchopulmonary dysplasia in the univariate analysis. Interestingly, nontraumatic intracranial hemorrhage was correlated with lower risk of epilepsy in univariate (OR−0.53, 95% CI [0.29–0.95], *p* = 0.034) and multivariable (OR = 0.24, 95% CI [0.13–0.45], *p* = 0.0001) analysis.

## 4. Discussion

Epilepsy is a significant global health concern, affecting millions of individuals worldwide. According to the World Health Organization (WHO), approximately 50 million people have epilepsy globally [23]. In our study, the prevalence of epilepsy among CP patients was carefully examined, with 44,901 out of 88,138 (51%) individuals identified as having epilepsy. Our prevalence corresponds with the previous range estimated by the published literature [4,5]. However, there may be some overestimation for the percentage as our cohort was derived from the NIS database with its hospital in-patient design. Previous studies faced a similar problem [4,24].

**Figure 1:**
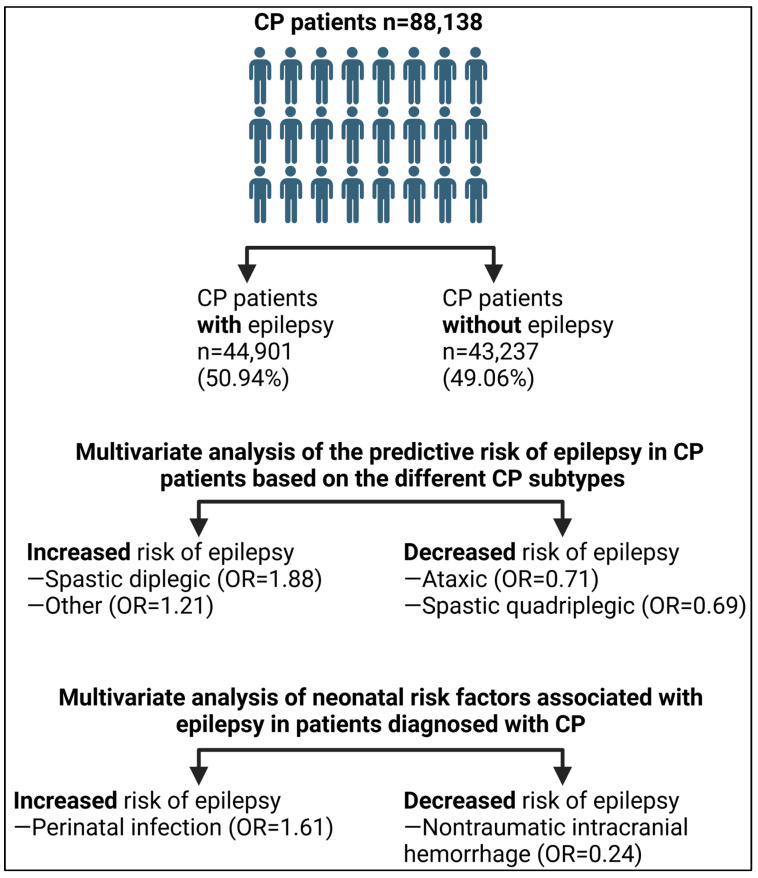
Graphical summary of the three specific objectives of the current research. CP: cerebral palsy; OR: odds ratio.

Figure 1 summarizes the three specific objectives of the current research, namely the prevalence of epilepsy, association between epilepsy and CP subtypes, and correlation between epilepsy and neonatal risk factors among CP patients.

Our study, encompassing 44,901 individuals out of a total of 88,138 from the NIS, stands as a significant contribution to the understanding of epilepsy in CP patients. To our knowledge, this investigation represents the largest population study focused on exploring the prevalence of epilepsy in CP patients and identifying associated risks and predictors. The sheer size of our cohort not only adds robustness to our findings but also enhances the generalizability of the results to a broader population of CP patients.

Our descriptive analysis highlights notable differences in racial composition, primary expected payer dynamics, and hospital settings between CP patients with and without epilepsy. These insights offer valuable considerations for tailoring clinical management strategies to address the distinct characteristics of each subgroup.

In our analysis, we observed a higher percentage of CP patients, with or without epilepsy, in urban areas as opposed to rural areas. This finding aligns with earlier research and suggests that families with children affected by CP may have a tendency to move to urban settings, potentially driven by the desire to access superior medical resources and more efficient healthcare professionals available in the more prosperous regions [17].

Most of our analyzed population had unspecified types of CP and epilepsy. We initially intended to limit our epilepsy correlations to the subgroup of patients with specified CP. However, upon careful review, we realized that focusing solely on specified CP types while excluding unspecified types would significantly reduce our cohort, hindering our ability to address the primary research question. Additionally, ICD-10 coding can be operator-dependent, with some healthcare providers using unspecified codes for reasons of simplicity, laziness, or due to lack of detailed information. Therefore, excluding unspecified types from analysis could lead to biased results and a less comprehensive understanding of the population. Future clinical and research implications highlight the need for healthcare providers to utilize all available resources, including thorough history-taking, physical examination, and radiological imaging, to accurately specify the type of CP and epilepsy. This is because accurate diagnosis is essential for establishing precise correlations.

Our analysis has revealed a substantial association of spastic quadriplegia, spastic diplegia, and other types of CP with epilepsy. This connection is closely tied to the extent of brain damage present in these specific subtypes of CP compared to others. It is well established that more extensive brain damage is characteristic of spastic quadriplegia and spastic diplegia, contributing to the observed heightened association with epilepsy [25,26,27]. Consistent with our findings, data from the literature indicates that epilepsy is more prevalent in spastic quadriplegic CP compared to other types, affecting over 30% of individuals [19]. This observation is supported by a high-quality systematic review and meta-analysis, which identifies spastic quadriplegic CP as the most significant factor contributing to epilepsy comorbidity in CP, with a prevalence of 62% based on 21 studies involving 2367 patients [4].

The link between increased brain damage and a higher likelihood of intellectual disability is a known and established risk factor for epilepsy [14]. Several studies underscore that as the severity of brain damage escalates, individuals with CP are more susceptible to intellectual disability, which in turn elevates the risk of developing epilepsy [4,15]. This correlation highlights the intricate interplay between the degree of brain injury, intellectual disability, and the propensity for epilepsy in individuals with cerebral palsy [28].

Magnetic resonance imaging (MRI) can be a valuable tool in determining the type and underlying causes of CP [29,30]. However, in our study, MRI information was not accessible. The literature pinpoints that white and gray matter lesions are more prevalent, whereas vascular insult lesions and brain malformations are less common [31]. Spastic CP is often linked to white matter damage, especially periventricular leukomalacia (PVL), followed by basal ganglia and gray matter injuries, with gray matter lesions more prevalent in severe cases [31]. Dyskinetic CP is likely to be associated with basal ganglia damage [31]. Ataxic CP, although less common, could be associated with cerebellar damage [31]. Early brain MRI can predict bimanual performance and cognitive outcomes, potentially identifying children who may benefit from targeted interventions [32].

Our study results are consistent with prior investigations, underscoring a significant correlation between infection and the onset of epilepsy in individuals with CP. Earlier research has specifically shown that maternal infection of the genitourinary system during pregnancy is associated with elevated risks of CP (adjusted hazard ratio [aHR] = 1.63, 95% CI: 1.34–1.98) and epilepsy (aHR = 1.27, 95% CI: 1.13–1.42) in the offspring [33]. These findings reinforce the notion that infection during pregnancy may play a crucial role as a potential risk factor for epilepsy development in CP patients [33]. Due to very small numbers of patients with neonatal aspiration (ICD-10 code: P24), congenital pneumonia (ICD-10 code: P23), respiratory distress (ICD-10 code: P22), and metabolic acidemia (ICD-10 code: P19), we could not create regression models for them in our analysis. Moreover, direct data about APGAR score was not available in ICD-10. Collectively, regarding conclusions about neonatal risk factors associated with epilepsy, we acknowledge the limitations of our data and emphasize the need for caution in interpreting these results due to the limited scope of data, small number of cases, and the absence of information on critical risk factors such as neonatal seizures. Notably, it has been shown that neonatal seizures amplify the risk of CP-related epilepsy three-fold [14].

Our research findings echo those of Douglass et al., who studied 966 children born before 28 weeks of gestation [34]. Of the 889 children evaluated at ages two and ten, 12.2% experienced at least one seizure, with 7.6% being diagnosed with epilepsy by age 10. The study concluded that the risk of epilepsy in this group was 7–14 times higher than in the general population, identifying shorter pregnancy durations as a primary risk factor [6,34]. Our study further supports these findings, highlighting a significant association between prolonged pregnancy duration (late labor) and a decreased risk of epilepsy.

Although not explored in our analysis due to lack of reliable data, maternal factors play a significant role in the development of epilepsy among children with CP. Sadowska and colleagues found maternal hypertension to be a substantial risk factor for epilepsy in CP patients (OR = 12.46) as well as for drug-resistant epilepsy (OR = 9.86) [6]. Additional risk factors include a positive family history of epilepsy, which significantly increases the likelihood of seizures in affected children [35]. Additionally, prolonged rupture of membranes before delivery has been associated with a heightened risk of epilepsy, suggesting that complications during pregnancy may have lasting impacts on neurological outcomes [35]. The mode of delivery is also critical; children born via lower segment cesarean section (LSCS) and those delivered by untrained birth attendants are at a greater risk for developing epilepsy [6,35]. These maternal factors underscore the importance of quality prenatal care and skilled attendance during childbirth to mitigate potential risks. Understanding these associations can inform healthcare providers in developing targeted interventions to reduce the incidence of epilepsy in children with CP. By addressing these maternal factors, including maternal health and delivery practices, it may be possible to improve the long-term outcomes for children affected by both conditions, highlighting the need for integrated care strategies that encompass maternal and child health.

Limitations should be considered, such as the use of hospital in-patient data from the NIS, potentially excluding non-hospitalized CP individuals and introducing selection bias. Moreover, the prevalence of epilepsy in the study might be overestimated due to the inpatient nature of the data source, which tends to capture more severe cases. The retrospective cross-sectional nature of the study limits establishing causation, and reliance on ICD-10 codes for CP diagnosis may lead to coding errors. Due to coding limitations and related very low numbers in the NIS database, we could not investigate the maternal-related risk factors that could affect the odds of developing CP among the offspring. Lack of information on CP severity, epilepsy medications, and other factors hampers nuanced analysis. The complex interplay of genetic, environmental, and individual factors contributing to epilepsy in CP patients requires further exploration. Further limitations comprise the relatively small fractions of patients with the examined variables, influencing the power of the analysis. Despite these limitations, the study is a significant contribution, highlighting the need for future research with comprehensive datasets and prospective designs to address these constraints.

## 5. Conclusions

This study aimed to provide comprehensive insights into the prevalence, risk factors, and predictors of epilepsy in individuals with cerebral palsy (CP) using a large sample size from the NIS database. The analysis revealed a significant association between specific CP subtypes—spastic quadriplegia and spastic diplegia—and epilepsy, highlighting how the severity of brain damage influences this relationship. Additionally, perinatal infection was found to be significantly associated with the occurrence of epilepsy among CP patients. Considering the acknowledged limitations of this research, the conclusions should be interpreted with caution. Prospective research is needed to validate these findings.

## Figures and Tables

**Table 1:** Demographic and healthcare characteristics of the included cerebral palsy patients.

Variable	All CP Patients n = 88,138	CP Patients with Epilepsy n = 44,901	CP Patients Without Epilepsy n = 43,237
Age	34 ± 22.51	28.4 ± 20.50	39.81 ± 23.03
Sex			
Male	49,568 (56.24%)	25,817 (57.50%)	23,751 (54.93%)
Female	38,570 (43.76%)	19,084 (42.50%)	19,486 (45.07%)
Year			
2016	20,530 (23.29%)	10,327 (23.00%)	10,203 (23.60%)
2017	21,785 (24.72%)	11,038 (24.58%)	10,747 (24.86%)
2018	22,628 (25.67%)	11,503 (25.62%)	11,125 (25.73%)
2019	23,195 (26.32%)	12,033 (26.80%)	11,162 (25.82%)
Type of CP			
Ataxic CP	324 (0.37%)	123 (0.27%)	201 (0.46%)
Athetoid CP	676 (0.77%)	326 (0.73%)	350 (0.81%)
Spastic hemiplegic CP	1308 (1.48%)	696 (1.55%)	612 (1.42%)
Spastic diplegic CP	6601 (7.49%)	2849 (6.35%)	3752 (8.68%)
Spastic quadriplegic CP	19,760 (22.42%)	13,530 (30.13%)	6230 (14.41%)
Unspecified CP	52,584 (59.66%)	23,793 (52.99%)	28,791 (66.59%)
Other CP	7953 (9.02%)	4246 (9.46%)	3707 (8.57%)
Types of epilepsy			
Focal idiopathic epilepsy	-	235 (0.52%)	-
Focal symptomatic simple partial	-	1459 (3.25%)	-
Focal symptomatic complex partial	-	2591 (5.77%)	-
Generalized epilepsy	-	433 (0.96%)	-
Other generalized epilepsy	-	3381 (7.53%)	-
Other recurrent seizures	-	3901 (8.69%)	-
Lennox–Gastaut syndrome	-	2091 (4.66%)	-
Epilepsy, unspecified	-	33,692 (75.04%)	-
Epilepsy due to external causes	-	52 (0.12%)	-
Race			
White	56,190 (63.75%)	26,449 (58.91%)	29,741 (68.79%)
Black	14,275 (16.20%)	7887 (17.57%)	6388 (14.77%)
Hispanic	12,398 (14.07%)	7648 (17.03%)	4750 (10.99%)
Asian or Pacific Islander	1827 (2.07%)	1027 (2.29%)	800 (1.85%)
Native American	563 (0.64%)	300 (0.67%)	263 (0.61%)
Other	2885 (3.27%)	1590 (3.54%)	1295 (3.00%)
Primary expected payer			
Medicare	34,555 (39.21%)	14,245 (31.73%)	20,310 (46.97%)
Medicaid	34,859 (39.55%)	21,074 (46.93%)	13,785 (31.88%)
Private insurance	15,540 (17.63%)	7888 (17.57%)	7652 (17.70%)
Self-pay	740 (0.84%)	293 (0.65%)	447 (1.03%)
No charge	38 (0.04%)	11 (0.02%)	27 (0.06%)
Other	2406 (2.73%)	1390 (3.10%)	1016 (2.35%)
ZIP income quartile			
1st–25th	261,20 (29.64%)	13,015 (28.99%)	13,105 (30.31%)
26th–50th	24,064 (27.30%)	12,318 (27.43%)	11,746 (27.17%)
51st–75th	21,124 (23.97%)	10,834 (24.13%)	10,290 (23.80%)
76th–100th	16,830 (19.10%)	8734 (19.45%)	8096 (18.72%)
Location/Teaching status of hospital			
Rural	5869 (6.66%)	2468 (5.50%)	3401 (7.87%)
Urban nonteaching	12,982 (14.73%)	5734 (12.77%)	7248 (16.76%)
Urban teaching	69,287 (78.61%)	36,699 (81.73%)	32,588 (75.37%)
Bed size			
Small	18,284 (20.74%)	8937 (19.90%)	9347 (21.62%)
Medium	22,540 (25.57%)	11,214 (24.97%)	11,326 (26.20%)
Large	47,314 (53.68%)	24,750 (55.12%)	22,564 (52.19%)
Hospital region			
Northeast	18,164 (20.61%)	9429 (21.00%)	8735 (20.20%)
Midwest or North Central	21,970 (24.93%)	10,787 (24.02%)	11,183 (25.86%)
South	30,092 (34.14%)	15,289 (34.05%)	14,803 (34.24%)
West	17,912 (20.32%)	9396 (20.93%)	8516 (19.70%)

CP: cerebral palsy; age is presented as mean ± standard deviation whereas all other variables are presented as number (%).

**Table 2:** The predictive risk of epilepsy in cerebral palsy patients based on the different cerebral palsy subtypes.

Type of CP	Univariate Analysis	Multivariable Analysis
Unspecified	0.57, [0.55–0.58], *p* < 0.0001	0.62, [0.60–0.64], *p* < 0.0001
Other	1.11, [1.06–1.17], *p* < 0.0001	1.08, [1.03–1.13], *p* = 0.001
Ataxic	0.59, [0.47–0.74], *p* < 0.0001	0.60, [0.48–0.75], *p* < 0.0001
Athetoid	0.89, [0.77–1.04], *p* = 0.150	-
Spastic hemiplegic	1.08, [0.97–1.21], *p* = 0.146	-
Spastic diplegic	0.71, [0.68–0.75], *p* < 0.0001	0.65, [0.62–0.68], *p* < 0.0001
Spastic quadriplegic	2.56, [2.48–2.65], *p* < 0.0001	2.37, [2.29–2.45], *p* < 0.0001

Data are presented as odds ratio, [95% confidence intervals], *p*-value. Multivariable analysis is adjusted for several factors.

**Table 3:** Neonatal risk factors associated with epilepsy in individuals diagnosed with cerebral palsy.

Variable	All CP Patients n = 88,138	CP Patients with Epilepsy n = 44,901	CP Patients Without Epilepsy n = 43,237	Univariate Analysis	Multivariable Analysis
Perinatal infection	202 (0.23%)	148 (0.33%)	54 (0.12%)	2.64, [1.94–3.61], *p* = 0.0001	1.61, [1.17–2.23], *p* = 0.004
Perinatal cardiovascular and respiratory disorders	1123 (1.27%)	597 (1.33%)	526 (1.17%)	1.00, [0.97–1.23], *p* = 0.135	-
Nontraumatic intracranial hemorrhage	48 (0.05%)	17 (0.04%)	31 (0.07%)	0.53, [0.29–0.95], *p* = 0.034	0.24, [0.13–0.45], *p* = 0.0001
Bronchopulmonary dysplasia	856 (0.97%)	459 (1.02%)	397 (0.88%)	1.11, [0.97–1.28], *p* = 0.115	-

CP: cerebral palsy; data are presented as odds ratio, [95% confidence intervals], *p*-value.

## Data Availability

Data were obtained from a publicly available database under the Hospital Cost and Utilization Project’s Nationwide Inpatient Sample, which can be accessed at the following link: https://www.hcup-us.ahrq.gov/db/nation/nis/nisdbdocumentation.jsp (accessed on 26 August 2024). [Hospital Cost and Utilization Project’s Nationwide Inpatient Sample] [https://www.hcup-us.ahrq.gov/db/nation/nis/nisdbdocumentation.jsp (accessed on 26 August 2024)] [NIS 2016–2019].

## Supplementary Materials

The following supporting information can be downloaded at: https://www.mdpi.com/article/10.3390/medicina60111809/s1, Table S1: Codes for data analysis and their source.
