# Correction: Alyoubi, R.A.; Abu-Zaid, A. Epilepsy in Cerebral Palsy: Unraveling Prevalence, Risk Factors, and Subtype Associations in a Large-Scale Population Study. *Medicina* 2024, *60*, 1809

**DOI:** 10.3390/medicina62050801

**Published:** 2026-04-22

**Authors:** Reem Abdullah Alyoubi, Ahmed Abu-Zaid

**Affiliations:** 1 Department of Pediatrics, Faculty of Medicine, King Abdulaziz University, Jeddah 22254, Saudi Arabia; 2 Department of Biochemistry and Molecular Medicine, College of Medicine, Alfaisal University, Riyadh 11533, Saudi Arabia

A few minor, unintentional errors were identified after publication; therefore, the authors wish to make the following corrections to this paper [1]:

**Error in Table**

In the original publication, there were a few minor errors in Table 2. These included the reversal of the adjusted odds ratios for the spastic diplegic and spastic quadriplegic subtypes in the multivariable analysis, as well as minor numerical corrections in few cells of Table 2. The corrected Table 2 appears below.

**Text Correction**

There were a few errors in the original publication. These relate to the incorrect description of the multivariable analysis in the Abstract and Results sections, arising from the unintentional reversal of the adjusted odds ratios.

A correction has been made to the Abstract, as follows:

Spastic quadriplegic CP showed the strongest association with epilepsy (adjusted OR = 2.37, 95% CI [2.29–2.45], *p* < 0.0001), underscoring the importance of subtype-specific considerations.

A correction has been made to Section 3 (Results), specifically Section 3.2 “Association Between Epilepsy and CP Subtypes Among CP Patients”, as follows:

In the multivariable analysis, the “spastic quadriplegic” CP subtype was associated with the highest risk of epilepsy (OR = 2.37, 95% CI [2.29–2.45]; *p* < 0.0001), whereas the “ataxic” CP subtype was associated with the lowest risk (OR = 0.60, 95% CI [0.48–0.75], *p* < 0.0001).

Additionally, the term “multivariate” has been corrected to “multivariable” throughout the manuscript.

The authors state that the scientific conclusions are unaffected. This correction was approved by the Academic Editor. The original publication has also been updated.

## Figures and Tables

**Table 2:** The predictive risk of epilepsy in cerebral palsy patients based on the different cerebral palsy subtypes.

Type of CP	Univariate Analysis	Multivariable Analysis
Unspecified	0.57, [0.55–0.58], *p* < 0.0001	0.62, [0.60–0.64], *p* < 0.0001
Other	1.11, [1.06–1.17], *p* < 0.0001	1.08, [1.03–1.13], *p* = 0.001
Ataxic	0.59, [0.47–0.74], *p* < 0.0001	0.60, [0.48–0.75], *p* < 0.0001
Athetoid	0.89, [0.77–1.04], *p* = 0.150	-
Spastic hemiplegic	1.08, [0.97–1.21], *p* = 0.146	-
Spastic diplegic	0.71, [0.68–0.75], *p* < 0.0001	0.65, [0.62–0.68], *p* < 0.0001
Spastic quadriplegic	2.56, [2.48–2.65], *p* < 0.0001	2.37, [2.29–2.45], *p* < 0.0001

Data are presented as odds ratio, [95% confidence intervals], *p*-value. Multivariable analysis is adjusted for several factors.
