# Supplementary material for: Epilepsy in Cerebral Palsy: Unraveling Prevalence, Risk Factors, and Subtype Associations in a Large-Scale Population Study
Source: Medicina (Kaunas). 2024 Nov 4;60(11):1809. doi: 10.3390/medicina60111809 (PMC11596662; doi:10.3390/medicina60111809)
Supplement: Supplementary file 1 [file medicina-60-01809-s001.zip › medicina-3280178-supplementary.pdf]

Supplementary Table S1. Codes for data analysis and their source.

**Variable**	**Source**	**ICD-10 code**
Cerebral palsy	I10_DX1/40	G80
Ataxic cerebral palsy	I10_DX1/40	G80.4
Athetoid cerebral palsy	I10_DX1/40	G80.3
Spastic hemiplegic cerebral palsy	I10_DX1/40	G80.2
Spastic diplegic cerebral palsy	I10_DX1/40	G80.1
Spastic quadriplegic cerebral palsy	I10_DX1/40	G80.0
Unspecified cerebral palsy	I10_DX1/40	G80.9
Other cerebral palsy	I10_DX1/40	G80.8
Focal idiopathic epilepsy	I10_DX1/40	G40.0
Focal symptomatic simple partial	I10_DX1/40	G40.1
Focal symptomatic complex partial	I10_DX1/40	G40.2
Generalized epilepsy	I10_DX1/40	G40.3
Other generalized epilepsy	I10_DX1/40	G40.4
Other recurrent seizures	I10_DX1/40	G40.8
Lennox-Gastaut syndrome	I10_DX1/40	G40.81
Epilepsy, unspecified	I10_DX1/40	G40.9
Epilepsy due to external causes	I10_DX1/40	G40.5
Perinatal infection	I10_DX1/40	P35-P39
Perinatal cardiovascular respiratory disorders	I10_DX1/40	P19-P29
Intracranial hemorrhage in newborn (other nontraumatic)	I10_DX1/40	I62
Perinatal bronchopulmonary dysplasia	I10_DX1/40	P27.1
Age	NIS Core	-
Sex	NIS Core	-
Primary expected payer	NIS Core	-
Race	NIS Core	-
Year	NIS Core	-
ZIP income quartile	NIS Core	-
Hospital bed size	NIS Hospital	-
